# Impact of microbiota on central nervous system and neurological diseases: the gut-brain axis

**DOI:** 10.1186/s12974-019-1434-3

**Published:** 2019-03-01

**Authors:** Qianquan Ma, Changsheng Xing, Wenyong Long, Helen Y. Wang, Qing Liu, Rong-Fu Wang

**Affiliations:** 1 Center for Inflammation and Epigenetics, Houston Methodist Research Institute, Houston, TX 77030 USA; 2 Department of Neurosurgery in Xiangya Hospital, Central South University, Changsha, 410008 China; 3 Institute of Biosciences and Technology, College of Medicine, Texas A&M University, Houston, TX 77030 USA; 4 Department of Microbiology and Immunology, Weill Cornell Medical College, Cornell University, New York, NY 10065 USA

**Keywords:** Gut microbiota, Central nervous system, Immune signaling, Neurological disorder, Glioma, Gut-brain axis

## Abstract

Development of central nervous system (CNS) is regulated by both intrinsic and peripheral signals. Previous studies have suggested that environmental factors affect neurological activities under both physiological and pathological conditions. Although there is anatomical separation, emerging evidence has indicated the existence of bidirectional interaction between gut microbiota, i.e., (diverse microorganisms colonizing human intestine), and brain. The cross-talk between gut microbiota and brain may have crucial impact during basic neurogenerative processes, in neurodegenerative disorders and tumors of CNS. In this review, we discuss the biological interplay between gut-brain axis, and further explore how this communication may be dysregulated in neurological diseases. Further, we highlight new insights in modification of gut microbiota composition, which may emerge as a promising therapeutic approach to treat CNS disorders.

## Introduction

Abundant and diverse microbial communities coexist in humans and mice. Majority of these microorganisms including bacteria, archaea, fungi, and viruses reside in human gastrointestinal tract, and are collectively referred as gut “microbiota” [1]. Studies on the symbiotic microflora trace back to almost 30 years [2]. Accumulating evidence suggests that microbiota are involved in the physiology and pathology of cellular organisms, and hence has implications in both health and disease [3]. Distinct microbial flora, which is inherited maternally at birth, changes due to our dietary habits and environmental signals [4–6]. The role of microbiota in various physiological activities, including in immune system, has been well established previously [7]. In addition, alterations in gut microbes in response to critical immune signaling contribute to the illnesses in intestine and distal organs, such as inflammatory bowel disease, autoimmune disease, and various types of cancer [8, 9].

The maturation and development of human central nervous system (CNS) is regulated by both intrinsic and extrinsic factors. Studies mostly from germ-free (GF) animals or animals treated with broad-spectrum antibiotics show that specific microbiota can impact CNS physiology and neurochemistry [10]. GF mice that are devoid of associated microflora exhibit neurological deficiencies in learning, memory, recognition, and emotional behaviors [11, 12]. They display variations in important neurotransmitters (e.g., 5-HT, NMDA, and BDNF) compared to conventional mice [13–15]. In humans, evidence for interplay between gastrointestinal pathology and neuropsychiatric conditions has been reported in conditions such as anxiety, depression, and autism [12, 16]. Furthermore, gut microbiota has been shown to modulate the development and homeostasis of CNS in context to immune, circulatory, and neural pathways [17]. In this review, we first discuss recent findings related to the interaction between gut microbiota and immune system, particularly key innate and adaptive immunity and signaling pathways. We then discuss the contribution of microbiota in CNS and pathogenesis of CNS disorders such as Parkinson’s disease (PD), Alzheimer’s disease (AD), multiple sclerosis (MS), and gliomas. Finally, we discuss the role of gut-brain interactions during development of nervous system and neurodegeneration, as well as potential approaches for treating CNS disorders.

### Interplay and reciprocal regulation between microbiota and immune system

The human immune system has evolved to maintain a symbiotic relationship between host and microbiota, and its disruption in dynamic immune-microbial interaction leads to profound effects on human health [18]. In this section, we discuss the interplay between resident microbiota and key immunological signaling, and implications of their relationship in CNS development and neurological diseases.

### Inflammasome signaling pathway

Inflammasome is an innate immune signaling complex, which is activated in response to diverse microbial and endogenous danger signals. To date, various pattern-recognition receptors (PRRs) in different families, including NLRP1, NLRC3, NLRP6, NLRP7, NLRC4 and AIM2, have been identified to play a role in inflammasome activation. Inflammasomes activation recruits ACS (apoptosis-associated speck-like protein containing a caspase recruitment domain) and the cysteine protease caspase 1 through caspase activation and recruitment domain (CARD) to induce the proteolytic cleavage of pro-caspase1 to generate mature and active caspase 1, which further process pro-IL-1β and pro-IL-18 to the final production of bioactive IL-1β and IL-18 proteins [19]. We identified NLRC5 as a key protein that negatively regulates NF-κB and type I interferon (IFN-I) signaling to control the homeostasis of innate immune system [20]. Earlier reports indicate that elevated levels of short-chain fatty acids (SCFAs) fermented by commensal microbiome activate NLRP3 inflammasome in gut epithelium through binding to GPR43 and GPR109A [21]. Furthermore, inflammasome activation leads to the release of IL-18, which contributes to the gut homeostasis and provides a protective role in colitis [21]. The protective effects of SCFAs in gastrointestinal graft-versus-host disease require GPR43-mediated ERK phosphorylation and activation of NLRP3 inflammasome [22]. NLRP6 inflammasome signaling plays an important role in modulation of microbiota. For example, NLRP6 deficiency leads to distorted colonization in intestinal microenvironment and possibly causes dysbiosis-driven diseases [23]. Further studies reveal that ASC, Caspase-1, and IL-18 knockout exhibit altered microbiota colonization as compared with that of wild-type mice. The inflammasome-mediated dysbiosis impacts a number of diseases [24]. Major depressive disorders are often associated with activated inflammasome and elevated levels of proinflammatory cytokines, such as IL-1β, IL-6, and IL-18 proteins [25, 26]. By contrast, inhibition of caspase-1 attenuates inflammation and anxiety-like behaviors and modulates the composition of gut microbiota. Anti-caspase-1-treated mice show increased flora of *Akkermansia* spp. and *Blautia* spp. related to the induction of Foxp3 regulatory T cells (Tregs), and suppression of IL-1β- and IL-6-mediated pathways [27]. Collectively, these studies indicate that gut microbiota modulate inflammatory response via inflammasome signaling to affect anxiety- and depressive-induced behaviors.

### Type I interferon signaling pathway

Type I interferon (IFN-I) is a pleiotropic and ubiquitous cytokine which plays an essential role in both innate and adaptive immunity and in the maintenance of host homeostasis. IFN-I is induced by pathogen-associated molecular patterns (PAMPs). Secretion of endogenous IFN-I depends on activation of several classes of PRRs, such as Toll-like receptors (TLRs), nucleotide-binding domain, and leucine-rich repeat containing gene family (NLRs) and RIG-I-like receptors (RLRs), and they play significant role in priming the host to various viral, bacterial, or tumor components [28, 29]. Upon activation, most TLRs recruit a common adaptor molecule, MyD88, which interacts with various downstream factors to activate NF-κB pathway [30]. IFN-I has also been shown to stimulate the maturation of DC and enhancement of cytotoxic T cells, which are crucial for immune responses against cancers [31]. Our previous study illustrated that MyD88-dependent IFN-I-stimulated maturation of plasmacytoid DCs was negatively regulated by SOCS1 [32]. Genetic ablation of SOCS1 caused robust production of IFN-α/β that led to potent adaptive immunity against lethal malaria infection [32]. Additional studies have suggested that IFN-I exhibits both positive and negative immunomodulatory functions in various human conditions. IFN-I provides no any therapeutic benefit in IBD, it may even exacerbate the disease [33]. By contrast, IFN-I regulates cell growth and induces apoptosis in several types of cancers including hematological malignancies and solid tumors [33]. Therapeutic application of IFN-I in autoimmune disorders (such as MS) have proved to be effective through the inhibition of inflammasome signaling [34]. Effects of IFN-I on inflammation and host hemostasis have been linked to the recruitment of Tregs [35, 36]. The role of IFN-I in modulation of microbiota has been extensively studied. For instance, two strains of *Lactobacillus acidophilus* have an ability to induce anti-viral responses via TLR2-dependent IFN-β in murine bone marrow-derived DCs [37]. Commensal lactic acid bacteria have been shown to trigger TLR3-mediated INF-β secretion by DCs in the intestine [38]. Metabolite produced by *clostridium orbiscindens* protects mice from influenza through augmentation of IFN-I signaling [39]. Protective microbiota-dependent IFN-I signaling is blocked by autophagy proteins [40]. Host IFN-I can also affect the composition of gut microbial communities, which suggests a bidirectional interaction between microbiota and IFN-I signaling [29]. These observations (regarding microbiota and IFN-I) point to the importance of synergistic factors in modulation of immune response to pathogenic challenges, and this potential interplay may also influence biological performance of CNS [41].

### NF-κB signaling pathway

NF-κB family of transcription factors contribute to both innate and adaptive immune responses and maintenance of immune system [42]. Our previous study identified dynamic K63-linked ubiquitination of NLRC5 which regulates NF-κB signaling and dynamically shapes inflammatory responses [20, 43]. Alterations in gut microbiota composition contribute to various inflammatory diseases via regulation of innate immunity, especially via NF-κB signaling [44]. Studies have shown that in ampicillin-treated mice, variations of succinate and butyrate leads to significant enhancement of NF-κB [45]. Moreover, the invasion by *Campylobacter jejuni* due to dysbiosis of intestine microbiome also resulted in activation of NF-κB due to secretion of various cytokines which stimulate different immune cells [46]. In contrast, another strain of microbiota, *Lachospiraceae* and its metabolites mediate protective function of NLRP12 in extreme inflammatory diseases by attenuating the activation of NF-κB/MAPK signaling and high fat diet-induced inflammasome activation [47]. Additional studies have revealed that the interaction between microbiota and NF-κB signaling is also responsible for CNS inflammation. For instance, the disturbance of gut microbiota induced by antibiotic treatment leads to inhibition of BDNF expression (in hippocampus) and activation NF-κB, which leads to severe neuroinflammation and anxiety-like behavior in animal models. In contrast, administration of *lactobacilli* alleviates CNS inflammation and mitigates anxiety-related symptoms [48]. Similarity, in a colitis model, elevated NF-κB is detected in intestines as well as hippocampal zone with cooperative expression of TNF-α, which leads to serious memory impairment. The restoration of unbalanced gut microbiota attenuated both colitis and amnesia [49].

## Microbiota influences in CNS components (gut-brain axis)

**Fig. 1:**
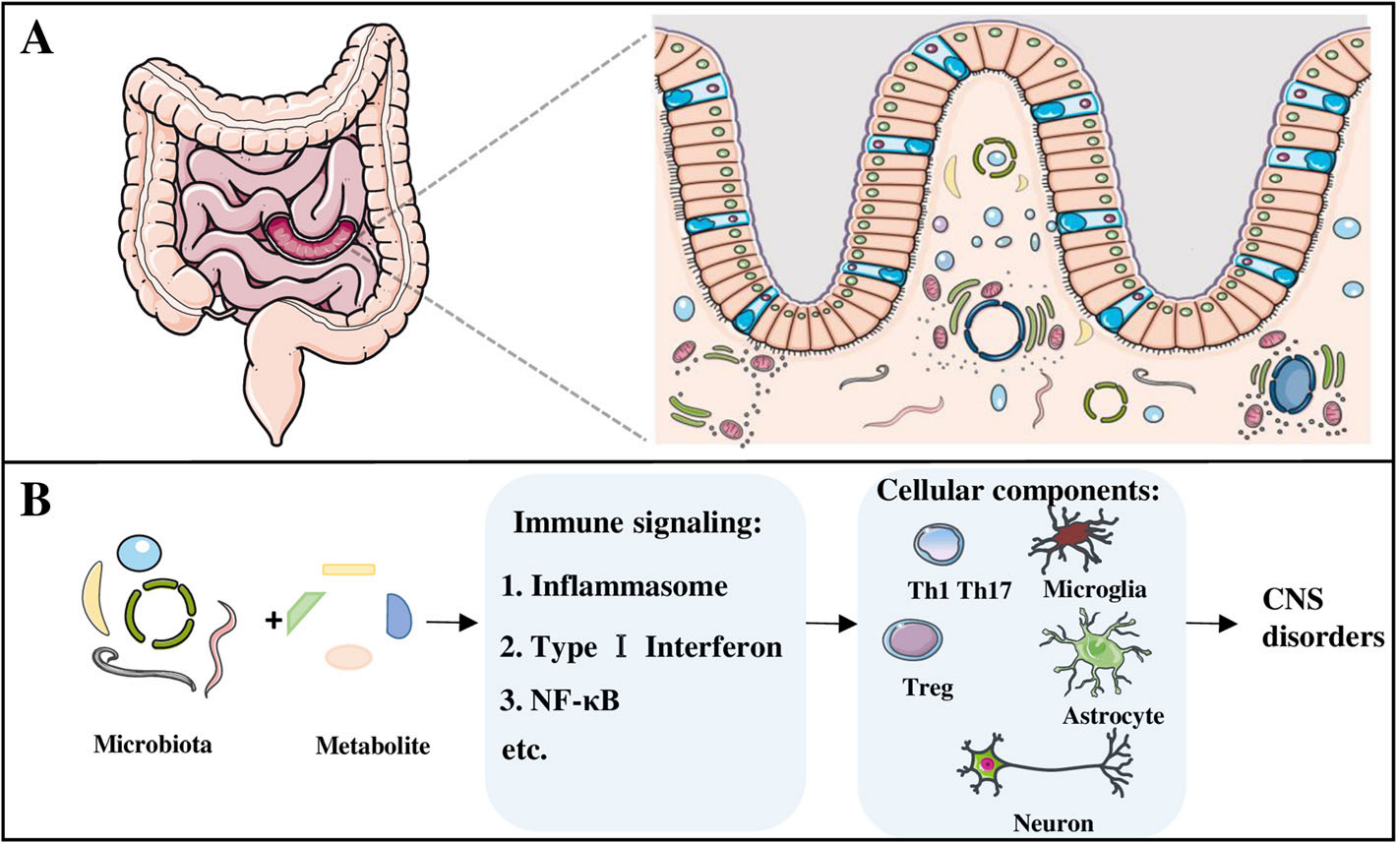
Microbiota and the gut-brain axis. **a** The majority of microorganisms reside in the gastrointestinal tract of human beings and impact wide range of physiological or pathological activities of the host. **b** The concept of “gut-brain axis” includes complicated direct and indirect interaction of gut microbiota and their metabolites with different cellular components in CNS through immunological signaling. Disruption of hemostasis in gut microbiota can lead to the alternations in CNS, resulting in the progression of various CNS disorders

**Fig. 2:**
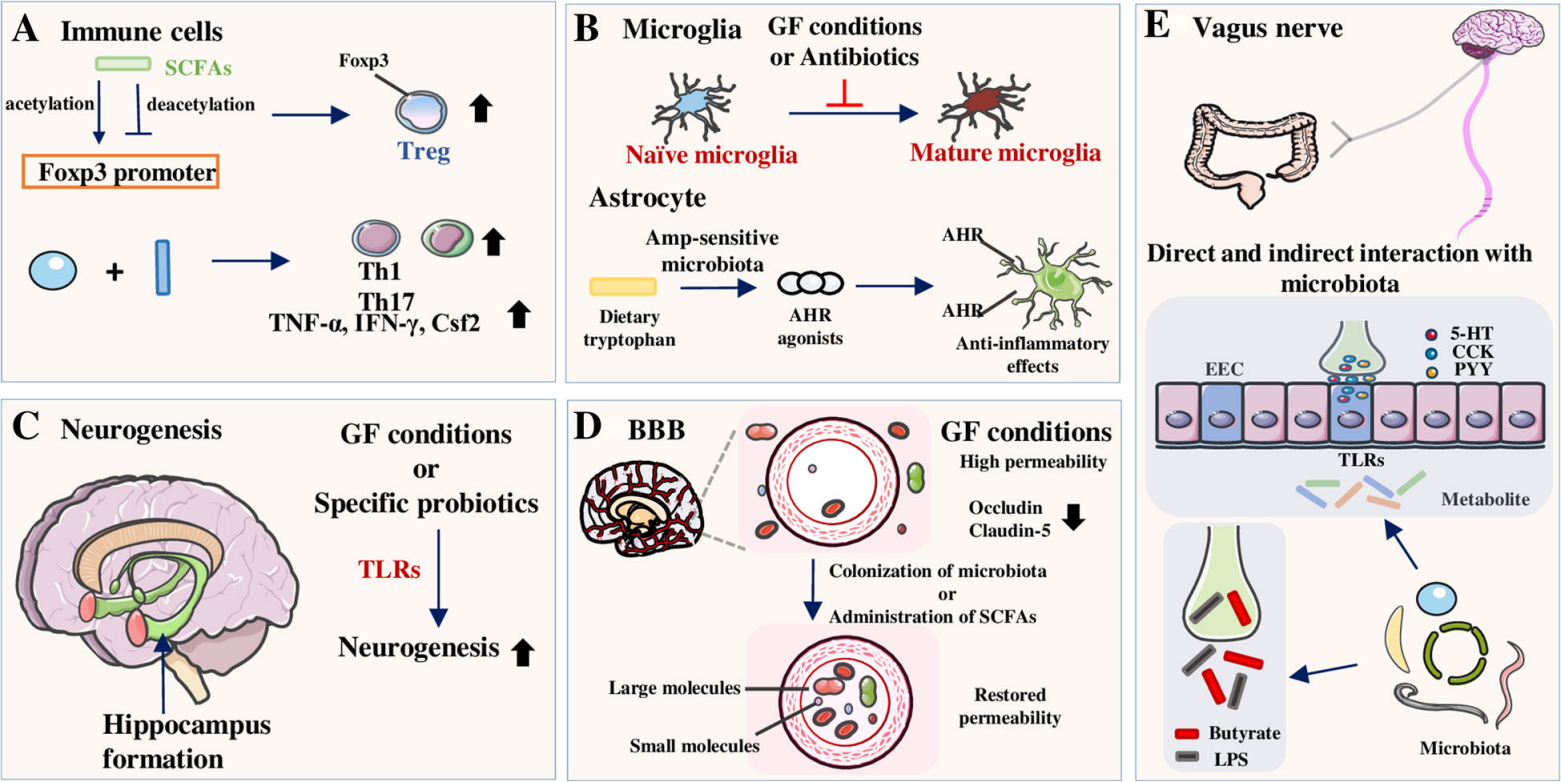
Influences of the gut microbiota on different components in the CNS. **a** The byproducts of bacterial metabolism in gut, SCFAs, are able to induce proliferation of Foxp3^+^ Tregs through histone-modification. Administration of specific strains of microbiota or metabolite promotes the development of Th1, Th17 cells, and other cytokines. **b** Gut microbiota contribute to the maturation progress of naïve microglia and the number of mature microglia decreases in the absence of microbiota while the total count of microglia remains the same. Amp-sensitive microbiota catalyze dietary tryptophan to AHR agonists which could bind to the AHR on astrocyte and induce anti-inflammatory effects. **c** Deletion of gut microbiota leads to neurogenesis in hippocampus in animals raised in GF conditions or treated with antibiotics. **d** BBB in GF mice are more permeable with decreased expression of tight junction proteins while the integrity of BBB could be restored by colonization of microbiota or supplementation of SCFAs. Vagus nerve is a critical component linking biological functions in gut and brain. Signals from gut could either directly interact with vagus nerve or indirectly through the mediation of EECs and hormonal factors

Gut-brain axis is used to define the relationship between microbiota and their interaction with brain, resulting in changes in CNS status (Fig. 1). A notable role of human digestive system in brain development has been proposed [15, 50]. Dysbiosis of microbial species may induce atypical immune signaling, imbalance in host homeostasis, and even CNS disease progression. In this section, we further discuss the cross-communication between commensal microorganisms and different components of CNS, and potential of immune signaling involved in this complex crosstalk (Fig. 2).

### Immune cells in CNS

Although CNS is frequently considered an immune-privileged site, the functional lymphatic vasculature (in dural meningeal membrane surrounding the brain) and the permeable brain–blood barrier (BBB) could serve as a gateway for signals transmission, thereby suggesting a role of immune cells in CNS during challenges [51, 52]. In addition to glial cells, the resident immune cells (such as macrophages, CD8^+^ T cells, Tregs, and other CD4^+^ T helper (Th) cell subsets) are actively involved in innate and/or adaptive immune responses [53–55]. Gut microbiota has been reported to promote different subsets of CD4^+^ T cells through antigen stimulation and activation of immune signaling pathways. For example, *Bacteroides fragilis* promotes the development of Th1 cells through polysaccharide A-dependent pathway [56], while *Clostridium* is shown to promote Treg cell differentiation [57]. In addition, segmented filamentous bacterium (SFB) stimulates the activation of Th17 and innate lymphoid cells [58–61], with specific bacterial antigens from SFB identified for gut Th17 cell activation [62]. Likewise, *Acinetobacter baumannii* and *Porphyromonas uenonis* also play an important role in promoting gut Th17 cells [63]. In experimental autoimmune encephalomyelitis (EAE) models, CD4^+^ Th cells play an important role in MS. Whereas IFN-γ-producing Th1 cells have pathogenic role in MS, IL-4- and IL-10-producing Th2 cells exhibit protective function [64]. Furthermore, Th17 cells are also involved in the pathogenesis of this disease, as mice lacking IL-23, a major cytokine for Th17 cells differentiation, are protected from EAE [64, 65]. Foxp3-expressing Tregs, which play critical roles in modulating inflammation in CNS, exert a suppressive function in EAE model via secretion of anti-inflammatory cytokines IL-10 and TGF-β [66].

Microbial metabolites have been well documented as activators of immune cells. As mentioned above, SCFAs activate inflammasome through GPR-dependent mechanisms to conduct suppressive functions in colitis [21], and the GPR-inflammasome reactions are also responsible for SCFA-induced differentiation of suppressive Tregs [67, 68]. Specifically, SCFAs induce proliferation of Foxp3^+^ Tregs via histone modifications, with increased acetylation and decreased deacetylation at Foxp3 promoter region [69, 70]. Furthermore, a large-scale production of butyrate and propionic acid from intestinal microbiota exhibits a protective effect in inflammatory reactions, with increased Tregs through Foxp3 promoter modification [69, 71]. In addition to Tregs, SCFAs are also reported to stimulate the production of retinoic acid in intestine, which inhibits Th17 cell differentiation and promotes Treg proliferation, thus contributing to the beneficial effects in neuroinflammation [72] and in preclinical model of MS as well [73]. Long-chain fatty acids (LCFAs), on the contrary, enhanced differentiation and proliferation of Th1 and Th17 cells, with increased mRNA expression of pro-inflammatory factors, e.g., TNF-α, IFN-γ, and Csf2, which further leads to a severe phenotype in MS animals [73]. Since an impaired BBB allows the transmission of these molecules, it is important to focus on the immune-regulating metabolites derived from gut and their roles in physiology and pathology of brain.

### Microglia and astrocytes

Microglia originate from yolk sac-derived erythromyeloid progenitors (EMPs; E9.0-E9.5), migrate to brain during development, and maintain until adulthood through local self-renewal [74]. Microglia have been reported to protect brain against various pathological conditions, through the involvement in immune response activation, phagocytosis, and cytokine production [75, 76]. In addition, microglia regulate synaptic transmission, synaptic pruning, and neuronal circuit formation, which are involved in brain development and homeostasis [75, 77–79]. Recent studies have shown that microbiome impacts the properties and function of microglia. For instance, with the absence of microbiota, microglia in GF mice not only display alteration in their morphological characteristics and gene expression profiles, but they also exhibit inhibition in their maturation state with an increased number of immature microglia in brain cortex [80]. Similarly, antibiotic treatment in normal mice is associated with increased naïve microglia, without obvious difference in total microglia number [80, 81]. Immature microglia are further suggested to functionally impair the immune activation and responses to challenges in GF mice, which is associated with downregulation of inflammatory factors and inhibited innate immune signaling pathways [16, 80]. Notably, microbial deficiency-associated immunosuppressive phenotype in GF mice can be normalized by postnatal administration of microbial SCFAs, suggesting that certain microbiota species can drive the maturation of microglia and maintain their homeostasis [80]. Furthermore, expression of GPR43 in innate immune cells mediates inflammatory responses by binding with SCFAs, and mice lacking GPR43 expression display severe defects in microglia (major alternations on dendrite length, number of segments, branching points, terminal points, and increased cell volumes), which are similar to the defects observed in GF mice [80]. Considering the intimate relationship between GPR43 and inflammasomes, maintenance of microglia-mediated immunological homeostasis may depend on the interplay between GPR43 and inflammasomes signaling. Recent studies have revealed that microglia also exhibit sex- and age-dependent responses to microbiota. For instance, microglia from male mice have more sensitivity to the deficiency of microbiome in embryonic stage, whereas in female mice, loss of microbiota leads to the most dramatic alterations in transcriptomic profiles during adulthood [82]. Dimorphic changes in microglial signatures establish a distinct connection between gut microbiota and sex-biased pathologies in CNS [82].

Astrocyte is the most abundant cell population in CNS and they outnumber neurons by almost fivefold [83]. Similar to microglia, astrocytes have multiple essential functions in the maintenance of CNS integrity, including control of blood perfusion in cerebrum, maintenance of brain–blood barrier (BBB) stability, regulation of ion gradient balance, and modulation of neuron or nutrient transmission [84]. Excessive activation of astrocytes is emerging as a vital mechanism underlying the production of neural cytotoxic or immune inflammatory substances, leading to CNS dysfunction and neurological disorders [85, 86]. Activation of astrocytes from their resting state is often affected by multiple factors within or outside of CNS, gut flora-mediated metabolites being one of them, which act on aryl hydrocarbon receptors (AHR) in animal models. Upregulated AHRs in astrocytes induce anti-inflammatory activity by restricting the recruitment and ability of neurotoxic immune cells through participation in IFN-I signaling [41]. Ampicillin-sensitive microbes in gut are able to catalyze conversion of dietary tryptophan to AHR agonists and contribute to the resistance against inflammation and protection of neurons from inflammatory attack [87, 88]. Additional studies have shown that mice treated with antibiotic ampicillin exhibit reduced AHR agonist levels and worse disease symptoms. However, mice supplemented with tryptophan metabolites show reduction in the severity of symptoms and pro-inflammatory molecules Ccl2 and Nos2 expression in astrocytes [41]. Distinct from the anti-inflammatory effects of specific microbes in gut, *Porphyromonas gingivalis*, one of the most common gram-negative bacterial species in oral chronic inflammatory diseases, stimulates astrocytes (via activation of TLR4 to produce increased levels of cytokines) and contributes to the neuroinflammatory lesions [89, 90]. Studies have shown that *P*. *gingivalis* is mediated by LPS which locates in the outer membrane of bacteria; activated *P*. *gingivalis* then trigger the toxic activation on astrocytes [91]. Collectively, these findings point to the species-specific effects of gut microbiota on astrocytes.

### Neurogenesis

During CNS development, the generation of neurons is affected by exposure to various environmental factors [15] while host microbiome also exhibits dynamic variation in its composition during brain maturation [92]. Previous studies suggest that the permeability of maternal-fetal interface allows regulators from gut bacteria to activate TLR2, which promotes fetal neural development and has potential impact on cognitive function during adulthood [93, 94]. Previous studies also point to the role of gut microorganisms in modulating and directing developmental progress of neurogenesis in CNS, and that this complex interaction mainly occurs in hippocampus [95, 96]. Hippocampal formation involves the limbic system, which is known for memory, and increased neurogenesis in this area weakens established memory but facilitates the encoding of new conflicting information in mice [97]. Critical role of microbiota in neurogenesis in hippocampus and its potential link with loss of memory comes from the studies conducted in GF mice. Proliferation of neurons at dorsal hippocampus is greater in GF mice than in conventional mice. However, post-weaning exposure of GF mice to microbial clones did not influence neurogenesis, suggesting that neuronal growth is stimulated by microbiota at an early stage [98]. The connection between microbiota and hippocampal neuronal generation is further strengthened by the findings that deficient neurogenesis can be counteracted by a probiotic combination of specific bacterial strains [99, 100]. As mentioned earlier, NF-κB signaling participates in microbiota-neuron axis. Studies indicate that microbiota disturbance leads to increased NF-κB activation and TNF-α expression with induced memory impairment in animal models, and the restoration of microbiota composition alleviates neuroinflammation in hippocampus and ameliorates relevant symptoms [49]. Additional studies are warranted to precisely define the specific pathways and microbial species that mediate neurogenesis and CNS health.

### Brain–blood barrier

As a selective barrier between brain and circulatory system, brain–blood barrier (BBB) develops during gestation and serves as a gateway for various signals from gut to brain. The BBB-permeable compounds usually have a low molecular weight, with little or no charge, and have lipid-soluble properties [101, 102]. Studies have shown that metabolic products in the intestines exhibit these characteristics, which enables their free access through BBB to modulate brain physiology [101, 103]. Due to the lack of gut microorganisms in GF mice, an intact BBB is disrupted with diminished expression of key tight junction proteins, i.e., occludin and claudin-5 in brain endothelium [104]. However, BBB permeability can be restored upon colonization of specific bacteria, such as *Clostridium tyrobutyricum*, which produce high levels of butyrate, or by the administration of bacterial fermentation products to GF mice [104]. Whereas greater BBB permeability is observed in sterile fetuses than in adults [105, 106], treatment with a low-dose of penicillin in young mice promotes BBB integrity and upregulates the expression of tight junction proteins via long-term alterations in gut microbiota [107]. Taken together, these studies suggest that BBB integrity is regulated by certain key components of microbiota, which in turn mediate the transmission of more microbial signals from gut to brain.

### Vagus nerve

Vagus nerve (VN) is a component in parasympathetic nervous system and a key route of neural communication between CNS and gut microbiota [108, 109]. VN actively participates in the bidirectional interactions between gut microbiota-brain to maintain homeostasis in both cerebrum and intestine. For example, perturbations of the nerve may cause either CNS dysfunction, e.g., mood disorders or neurodegenerative diseases, or gastrointestinal pathologies, such as inflammatory bowel disease and irritable bowel syndrome [110–112]. Previous studies have indicated that vagal efferent fibers regulate the responses to environmental or pathophysiological conditions in gastrointestinal system via the release of neurotransmitters [113, 114]. A minor inappropriate activation of VN results in excessive activation and elevation of neurotransmitters, thereby impairing the digestive process and influencing gastric motility [112, 115]. Moreover, immune regulatory effects of VN on local immunity and intestinal permeability have also been observed. Studies have established that the activation of M1 macrophages and increased levels of proinflammatory cytokines induced by abdominal surgery are alleviated by electrical vagal stimulation, which might relieve inflammatory reactions after surgery and improve postoperative recovery [116]. Furthermore, the stimulation of VN by electro acupuncture promotes the expression and proper localization of tight junction proteins, thus decreasing intestinal permeability and exerting protective effects in intestinal epithelium barrier [117, 118].

Microbes rely on other types of cells located in the epithelium to transmit physiological signals from gut to brain [119]. Enteroendocrine cell (EEC) is one subtype of epithelial cells (less than 1%), which secrete various factors in metabolic processing of dietary nutrients [120, 121]. Due to the anatomical position and function, EECs communicate with gut microbiota to send output signals in forms of hormones to afferent neurons [122, 123]. The production of hormones such as 5-hydroxytryptamine (5-HT), cholecystokinin (CCK), and peptide YY (PYY) by EECs is stimulated by bacterial metabolites via TLRs expressed on the surface of EECs [123, 124]. These hormonal mediators are involved in further activating neural afferent fibers by binding to chemoreceptors [125, 126]. Additionally, a study found that signal transduction from gut can be completed by direct interactions with vagal afferent fibers in a specific subset of EECs [127]. Monosynaptic tracing revealed a functional synapse between special EECs with vagal nodose neurons, thus connecting the intestinal lumen with CNS and neurotransmitter glutamate (inside this synapse), which transduces signals to vagal neurons and completes the neuroepithelial circuit [127]. Modulation of VN by gut flora is further supported by the observation that oral administration of *Campylobacter jejuni* promotes the activated state of neurons in nucleus tractus solitarius, as the first intracranial entrance of vagal afferents [128, 129]. On the contrary, another report indicates that vagotomized mice treated with *Lactobacillus rhamnosus* show minimal improvement in anxiety- and depression-related behaviors, with no change in the expression of GABA receptors in brain [108]. A Swedish register-based matched-cohort human study provides a suggestive evidence for a potentially protective effects of truncal, but not of selective vagotomy in PD development, supporting the hypothesis that original pathological signals of PD start from peripheral tissues and later spread to CNS by VN-mediated mechanisms [130, 131]. Additional studies have shown that VN stimulation is widely used an as effective treatment method for intractable epilepsy and to improve the related mental symptoms [132, 133]. Thus, administration of probiotics to modify VN function could be a promising strategy in the future for the treatment of CNS disorders.

## Microbiota and CNS disorders

Since microbiota influences CNS through various immunological pathways (such as inflammasome, IFN-I, and NF-κB), it is reasonable to consider its contribution in progression of various neurological disorders. Here, we discuss the involvement of microbiota in neuroinflammation or neurodegenerative pathologies and discuss potential therapeutic approaches for the treatment of various diseases.

### Multiple sclerosis

Multiple sclerosis (MS) is an inflammatory disease characterized by the immune-mediated demyelination of neural axon. Loss of myelin results in varying degrees of distinct neurological disorders, including motor, sensory, visual, autonomic, and cognitive impairment [134–136]. Abnormal CD4^+^ T cell-related immune responses, especially the secretion of proinflammatory cytokines from hyperactive Th1 and Th17 cells, lead to the infiltration of various immune cells in CNS, initiating an immunogenic attack against myelin sheath surrounding neurons [137, 138]. Poor immunosuppressive activities of Tregs in MS patients may also worsen the aberrant autoimmune reactions [139, 140]. It has been suggested that MS pathogenesis originates in the immune system, with significant contributions of both genetic and environmental factors [141]. Since gut microbiota regulates both innate immune signaling and certain physiological processes in CNS, it has also been speculated to control the pathogenesis of MS [142].

EAE model, an autoimmune animal model induced by CD4^+^ T cells, is widely used to investigate MS [143], and studies have suggested that oral administration of antibiotics significantly reduces disease severity as it enhances the recruitment and proliferation of Foxp3^+^ Tregs [144]. Germ-free mice have been reported to show highly attenuated development of EAE, possibly due to increased Treg cells, while IFN-γ and IL-17-producing Th1 and Th17 cell population decreases compared to those in conventionally maintained mice [145]. Furthermore, segmented filamentous bacteria, which induce Th17 cell differentiation, are responsible for the development of EAE [58, 144]. The symptoms are ameliorated in GF mice harboring segmented filamentous bacteria alone, accompanied by restored levels of Th17 cells in CNS [58]. Potential for gut dysbiosis in disease-promoting conditions has also been discussed in MS patients. In a clinical study, in which 71 untreated MS patients were compared with healthy controls, elevated levels of specific taxa in microbiomes (e.g., *Akkermansia muciniphila* and *Acinetobacter calcoaceticus*) are observed in MS patients. Transplantation of these bacteria from patients with MS into GF mice leads to the exacerbation of EAE via increased proinflammatory T cell response and weakened Treg response [146]. Similar results are obtained in a study in which microbes from MS patients with pathogenic components aggravated MS-related symptoms in a transgenic mouse model [147]. Additional studies have shown that microbial taxa of pediatric patients with MS exhibit greater pro-inflammatory trend when compared to that of healthy children, and depletion of certain flora components in children with MS may be linked to an increased risk of relapse [148, 149]. In addition, treatment of MS by probiotic VSL3 induces enrichment of specific microbial species in intestine and inhibits peripheral inflammation mediated by monocytes. The anti-inflammatory responses disappear after discontinuation of VSL3 [150]. Collectively, these findings provide a basis for future studies pertaining to the microorganisms and pathways involved in the progression of MS. Modification of microbiota or subtle dietary changes could potentially contribute in the treatment of MS.

### Parkinson’s disease

Parkinson’s disease (PD) is a common neurodegenerative disorder which exhibits multifactorial motor symptoms, including tremor, muscular rigidity, slowness of movement, and gait abnormality [151]. Complex genetic and environmental factors are involved in the initiation and development of PD, which presents a major clinical challenge for disease treatment, as symptom relief becoming less effective during disease progression [152]. Principal pathology of PD is characterized by loss of dopaminergic neurons in substantia nigra, accompanied with the accumulation of α-synuclein and deposition of Lewy bodies in remaining neurons [153]. Emerging evidences suggests that α-synucleinopathy is initiated in enteric nervous system before it occurs in CNS during the early stages of disease, which is associated with some specific digestive symptoms [154, 155]. This has been documented in mice transfected with human wild-type α-synuclein, which exhibit constipation and impaired colonic motor function [156]. In this case, signals in PD might spread from gut to brain, and focus on the early pathogenesis or symptoms in intestinal tract may improve our understanding of the initiation of this disease.

Neurological diseases are historically studied within CNS; however, recent studies have implicated that peripheral influences in the onset and progression of diseases impact the brain [157]. Evidence from a study of α-synuclein overexpressing (ASO) mouse model of PD suggests a role of microbiota in the evolution of this disease [158]. ASO mice under a germ-free environment or treated with antibiotics show increased inhibition of PD-associated neuropathology compared with the mice from regular housing condition, whereas depletion of gut microorganisms in young ASO mice inhibited the progression of PD in adulthood. Furthermore, the symptom-free state could be preserved by either colonization via feces from conventional mice or oral administration of bacterial metabolites to these germ-free mice. In addition, activated expression of TLRs also contributes to the inflammation and neurodegeneration in PD. [159] Specifically, TLR4 is reported to interact with misfolded α-synuclein, and trigger downstream microglial reactions, production of proinflammatory cytokine, and oxidative stress promotion [160]. Similarly, TLR2, another molecule in TLRs family, has been found to be effective agonist of extracellular α-synuclein released by neuronal cells. Combination of TLR2 with α-synuclein promotes downstream neurotoxic signals involving MyD88 and NF-κB, resulting in the production of TNF and IL-1β [161, 162]. Notably, patients with PD exhibit higher exposure to gut microbiota due to their impaired intestine function. Consistent interconnection between microbial metabolism and TLRs induces elevated local inflammation and dysfunction in clearance of α-synuclein deposition, which synergistically contribute to the neurodegeneration of PD. [159] Moreover, colonization of germ-free mice via feces from PD patients led to more physical impairments than those observed using feces from healthy controls [158]. Further, a higher abundance of putative proinflammatory bacteria and reduced numbers of bacteria with anti-inflammatory properties were observed in fecal samples and sigmoid mucosal biopsies from patients with PD, corresponding to the inflammation-related misfolding of α-synuclein and pathology of PD in CNS [163]. Bacterial composition within the intestinal tract clearly influences PD, and other studies have provided detailed evidence for a role of gut dysbiosis in the disease. Severity of symptoms, including postural instability and gait abnormality, is associated with alterations in the abundance of certain species of *Enterobacteriaceae* [164, 165]. Besides, a reduction of *Lachnospiraceae* leads to a more severe impairment of motor and nonmotor symptoms in PD patients [165]. Considering the metabolites from gut microbiota could reveal or regulate the physiological status of both host and immune system, such as metabolites SCFAs [166, 167], explicit relationships between microbiota, and the development of PD may provide us novel biomarkers and mechanistic insights to this disease, and antibiotics or probiotics targeting these relationships may serve as an effective treatment strategy.

### Alzheimer’s disease

Alzheimer’s disease (AD) is a chronic and irreversible neurodegenerative disease and the most common form of dementia in the elderly. Patients with AD display serious CNS dysfunctions in learning, memory, and behavioral issues, leading to serve disability in daily activities [168, 169]. AD is characterized by loss of neurons and progressive impairments in synaptic function, accompanied with a deposition of amyloid-β (Aβ) peptide outside or around neurons, together with an accumulation of hyper-phosphorylated protein tau inside cortical neurons [170–172]. Aβ overload and tau aggregation foster microtubule destabilization, synaptic deficiency, disruption of Ca2^+^ homeostasis in neurons, and ultimately neuronal apoptosis [173, 174]. Despite recent advances in research, the mechanisms underlying AD are unclear, and current therapies targeting Aβ only provide modest symptom relief [175].

Previous studies have indicated that pathogenesis of AD is associated with peripheral infectious origin, which can cause neuroinflammation in CNS [176, 177]. Typical characteristics of Aβ and tau deposition in AD are directly linked with herpes simplex virus type 1 (HSV1) infection in mice. Virus infection selectively upregulates the expression of gene encoding cholesterol 25-hydroxylase (CH25H), which is critical for modulation of both AD susceptibility and Aβ production [178, 179]. Further, past studies have established the potential mechanistic connections between AD pathology and other types of infections, such as *spirochaete*, *fungus*, and *Chlamydia pneumoniae* infections [180–182]. Likewise, recent studies have implicated gut microbiome as a vital factor in the etiology of AD. Detection of metabolic molecule from microbiota in cerebrospinal fluid of AD patients, which is associated with biomarkers of AD (phosphorylated tau and phosphorylated tau/Aβ_42_), indicates the involvement of gut microbiota in pathogenesis of AD [183]. In an Aβ precursor protein (APP) transgenic mouse model, APP-mutant germ-free mice have decreased cerebral Aβ amyloid pathology when compared with APP mice in control conditions. Anti-Aβ effects could be blocked by reconstruction of these germ-free APP mice with microbiota from conventional mice [184]. Moreover, long-term broad-spectrum antibiotic treatment also reduces Aβ deposition and improves the neuropathological phenotype of mice with AD [185]. When comparing fecal microbiomes and fecal SCFAs between AD suffering mice and WT mice at different ages, dramatic elevations in *Verrucomicrobia* and *Proteobacteria*, as well as significant reductions of *Ruminococcus* and *Butyricicoccus* are observed in AD mice, suggesting altered microbiota composition and diversity, whereas the reduced level of SCFAs further indicates the alterations in many metabolic pathway [186]. Previous study has also shown that activated microglia contribute to pathology of AD by inhibiting Aβ clearance and increasing Aβ deposition [187]. Elevated deposition of Aβ results in the release of various proinflammatory mediators through microglia, including iNOS, ROS, COX2, and NF-κB, thereby causing neuroinflammation in AD pathogenesis [187]. Taken together, these results indicate that specific species of gut microbiota activate Aβ signaling pathways and contribute to the pathogenesis of AD. As the role of more microbial taxa are evaluated, nutritional interventions or probiotics/antibiotics may become novel therapeutic strategies to restrain the progression of AD.

### Gliomas

Glioblastoma is one of the most malignant tumors with dismal mortality rates [188]. Therefore, novel therapeutic agents and approaches are necessary to combat this deadly disease. Recent studies demonstrate the potential role of microbiome in immuno-oncology, with particular emphasis on the immune checkpoints [189]. Further, commensal microbiota have been shown to play therapeutic role in several tumor types [189, 190], with an unexpected observation of an anti-tumor role of *Bifidobacterium* in cooperation with innate immune system and PD-L1 blockade. These studies demonstrate that oral administration of *Bifidobacterium* in mice abolishes tumor outgrowth by inducing pathways involving the maturation of DCs, stimulation of tumor-specific CD8^+^ T cells, recruitment of other immune cells, and activation of type I interferon signaling [191]. Similarly, when analyzing stool samples from patients with metastatic melanoma, *Bifidobacterium longum*, *Collinsella aerofaciens*, and *Enterococcus faecium* have increased abundance in subjects that responded to a PD-1 inhibition with therapeutic antibodies, suggesting that certain microbial taxa in gut may provide supportive role to enhance the effects PD-1 blockade [192]. Furthermore, transplantation of fecal materials from responders into germ-free mice has been shown to improve the responses to PD-1 blockade and control tumor growth [192]. Consistently, antibiotic treatment before/during PD-1 blockade therapy impairs the treatment efficacy and overall survival time in patients with epithelial cancers [193]. Another recent study further shows that application of gut microbiota from the responders to GF mice has clear benefits by enhancing checkpoint blockade *in vivo* [194]. Besides, dependence of another critical immune checkpoint molecule CTLA-4 on microbiome has been reported to further demonstrate the influence of specific microbiota composition (*Bacteroides thetaiotaomicron* and/or *B*. *fragilis*) to the efficacy of CTLA-4 blockade therapy in mice and patients, through elevated IL-12-dependent Th1 immune responses [195].

Previous studies have clearly shown that the benefits of both chemotherapy and radiation therapy on tumor progression could be compromised by antibiotic treatment. For instance, anti-cancer activity of an immunostimulatory alkylating agent, cyclophosphamide, is limited in antibiotic-treated tumor-bearing mice due to lack of relevant Th1 and Th17 immune responses in spleen [196]. Further studies confirm that the presence of key bacterial species, *Enterococcus* and *Barnesiella*, is both necessary and sufficient to mount effective immune responses (such as induction of memory Th1 and pathogenic Th17 responses as well as increases in tumor-specific CD4^+^ and CD8^+^ T cells) at tumor location, thereby compensating for limited efficacy of cyclophosphamide [197]. Total body irradiation (TBI) has been shown to efficiently control tumor recurrence by multiple mechanisms and it maximizes the efficacy of adoptively transferred CD8^+^ T cells. Interestingly, antibiotic treatment or neutralization of serum LPS has been shown to weaken the beneficial effects of TBI on tumor regression, while LPS administration to non-irradiated mice enhances the number and function of transferred CD8^+^ T cells, indicating that microbiota facilitates the effects of TBI via metabolite of LPS [198, 199]. Further, it has been shown that CK (a metabolite of ginseng saponin) is produced by intestinal bacteria after oral administration of ginseng, which reduces the migration and invasive capabilities of glioma cells in vitro by inhibiting downstream SDF-1 and CXCR4 signaling [200]. Therefore, based on the emerging evidences which show that specific microbial taxa augment the effects of various therapeutic modalities against tumors, we could speculate that microbiota could be used to maximize the effects of current antitumor approaches and could even be used as biomarkers to predict prognosis and treatment responses in glioma patients [201]. However, additional studies are required to determine the detailed function of certain microbial components for glioma treatment.

## Conclusion

Due to complicated etiologies and lack of reliable biomarkers in humans, effective treatment strategies for CNS diseases have been of great interest. The concept of gut-brain axis is being actively explored, and many studies have confirmed that alterations in gut microbiota composition are associated with certain clinical conditions. Existence of a biological link among microbiota, immune signaling, and CNS indicates that both neurological and immunological activities in brain could be determined either directly by microbial metabolites or indirectly by microbiota-derived systemic signals. The applications of therapeutic modulators have already shown promising results in various mood disorders, such as autism and depression. However, as the details of gut-brain axis are still unclear, it is critical for future studies to clarify specific mechanisms by which gut microbes contribute to the progression or regression of certain pathological conditions. These studies may provide a basis for advanced therapeutic approaches, along with current therapeutic modalities as well as the identification of novel biomarkers, for early diagnosis and intervention of CNS disorders.

## Abbreviation

5-HT: 5-Hydroxytryptamine
AD: Alzheimer’s disease
AHR: Aryl hydrocarbon receptors
APP: Aβ precursor protein
ASO: α-Synuclein overexpressing
Aβ: Amyloid-β
BBB: Brain–blood barrier
CCK: Cholecystokinin
CH25H: Cholesterol 25-hydroxylase
CNS: Central nervous system
EAE: Experimental autoimmune encephalomyelitis
EECs: Enteroendocrine cells
EMPs: Erythromyeloid progenitors
GF: Germ-free
HSV1: Herpes simplex virus type 1
IFN-I: Type I interferon
LA: Lauric acid
LCFAs: Long-chain fatty acids
MS: Multiple sclerosis
NLRs: Nucleotide-binding domain and leucine-rich repeats
PAMPs: Pathogen-associated molecular patterns
PD: Parkinson’s disease
PRRs: Pattern-recognition receptors
PYY: Peptide YY
RLRs: RIG-I-like receptors
SCFAs: Short-chain fatty acids
SFB: Segmented filamentous bacterium
TBI: Total body irradiation
Ths: T helper cells
TLRs: Toll-like receptors
Tregs: Regulatory T cells
VN: Vagus nerve
